# Gene Therapy in Diabetic Retinopathy and Diabetic Macular Edema: An Update

**DOI:** 10.3390/jcm14093205

**Published:** 2025-05-06

**Authors:** Maricruz Odio-Herrera, Gloriana Orozco-Loaiza, Lihteh Wu

**Affiliations:** Asociados de Mácula Vitreo y Retina de Costa Rica, San José 60612, Costa Rica; mariodioh@gmail.com (M.O.-H.); glorianaorozco.06@gmail.com (G.O.-L.)

**Keywords:** diabetic retinopathy, diabetic macular edema, PDR, VEGF, vascular endothelial growth factor, gene therapy, 4D-150, RGX-314, Ixo-vec, ADVM-022, gene therapy associated uveitis

## Abstract

Diabetic retinopathy (DR) is one of the leading causes of preventable blindness worldwide. It is characterized by a spectrum of disease that spans mild non-proliferative diabetic retinopathy (NPDR) all the way to neovascular glaucoma and tractional retinal detachment secondary to proliferative diabetic retinopathy (PDR). Most eyes with DR remain asymptomatic unless vision-threatening complications, such as diabetic macular edema (DME) and/or PDR, develop. Current treatment options include laser photocoagulation and/or anti-VEGF intravitreal injections. Patients under treatment with anti-VEGF agents usually require constant monitoring and multiple injections to optimize outcomes. This treatment burden plays a key role in suboptimal adherence to treatment in many patients, compromising their outcomes. Gene therapy has emerged as a promising therapeutic option for DR. The mechanism for current trials evaluating gene therapies for DR consists of delivering transgenes to the retina that express anti-angiogenic proteins that inhibit VEGF. Preliminary results from the SPECTRA (4D-150) and ALTITUDE (ABBV-RGX-314) studies are promising, demonstrating an improvement in the diabetic retinopathy severity score and a reduction in the treatment burden. In contrast, the INFINITY (ADVM-022) trial was complicated by several cases of severe inflammation and hypotony that led the sponsor to discontinue further development of this product for DME.

## 1. Introduction

The International Diabetes Federation (IDF) recently estimated that 11.1% of the worldwide population between 20 and 79 years of age were affected by DM. This represents 589 million individuals, of which about 43% (252 million) were undiagnosed. Most of these undiagnosed persons live in low- and middle-income countries. Current projections suggest that, by 2050, the global number of diabetic patients will increase to 853 million. Low and middle-income countries will bear the brunt of this increase [1]. All of these individuals will be at risk of developing diabetic retinopathy (DR).

Untreated DR typically leads to blindness [2]. DR constitutes one of the leading causes of blindness in the working-age population and has a considerable economic impact on society, particularly on healthcare systems [3,4,5,6,7]. The development of DR is strongly associated with poor hyperglycemic control, and higher levels of HbA1c are associated with disease progression. DR is a progressive condition characterized by microvascular alterations that lead to retinal ischemia, an increase in retinal vasopermeability, retinal neovascularization, and macular edema [8,9]. Vision-threatening complications from DR include diabetic macular edema (DME) and proliferative diabetic retinopathy (PDR). DME is characterized by an excessive vascular permeability that leads to an extravasation of plasma constituents and accumulation of extracellular fluid in the inner retina [10]. PDR is characterized by retinal neovascularization and tractional retinal detachment [11].

At the molecular level, chronic hyperglycemia leads to several biochemical alterations that cause tissue hypoxia and vascular endothelial growth factor (VEGF) upregulation and secretion. Increasing tissue hypoxia leads to the upregulation of hypoxia-inducible factor 1 (HIF-1), which is an upstream transcriptional regulator of VEGF [12,13]. Once VEGF is produced by the ischemic retinal cells, it diffuses toward the retinal vascular endothelial cells. The retinal endothelial cells express several VEGF tyrosine kinase receptors on their surfaces. VEGF receptor 2 (VEGFR-2) is the major mediator of the angiogenic and vascular permeabilizing effects of VEGF. The binding of VEGF to VEGFR-2 leads to its dimerization and the autophosphorylation of intracellular tyrosine residues, which initiates the signal transduction that leads to endothelial proliferation, endothelial survival, transcriptional activation, endothelial migration, and vascular permeability [11,13,14].

Clinically, these biochemical alterations are initially just manifested as microaneurysms. As the degree of metabolic abnormalities increase with time, other findings that include intraretinal hemorrhages, cotton wool spots, venous beading, and neovascularization start to appear. The diabetic retinopathy severity scale (DRSS) was initially designed for the Diabetic Retinopathy Study (DRS) and was further modified for the Early Treatment of Diabetic Retinopathy Study (ETDRS) to describe and classify the severity of DR [15,16]. Typically, eyes with mild to severe non-proliferative diabetic retinopathy (NPDR) are asymptomatic unless DME is present. The development of PDR and visual loss correlate with worsening in the DRSS score [16,17].

The treatment of diabetic patients has traditionally been centered on fixing the complications of diabetic retinopathy, namely DME and PDR. For many decades until the 2010s, macular laser photocoagulation (MLP) was the treatment of choice for DME [18]. Currently, intravitreal anti-VEGF drugs have replaced MLP as the standard of care for DME [19,20,21]. Similarly, the standard of care for PDR for nearly half a century was panretinal photocoagulation (PRP) [11]. Currently, both PRP and VEGF inhibitors are used to manage PDR. Recent results from randomized clinical trials comparing ranibizumab and aflibercept with PRP have shown that anti-VEGF drugs cause the regression of intraocular neovascularization [22,23,24,25]. Patients under treatment with anti-VEGF agents usually require constant monitoring and multiple injections. The frequency of these visits and injections constitutes a major burden and may influence treatment compliance and lead to worse outcomes. Even short delays in treatment can have detrimental effects on visual function. If these patients somehow interrupt their treatment, they are at a high risk of developing irreversible blindness [11,26,27,28].

In eyes with DME, it was noted that eyes treated with multiple anti-VEGF injections had an improvement in their DRSS score [29]. Following 2 years of monthly injections, 36% of eyes treated with ranibizumab compared with 5% of sham-treated eyes experienced an improvement of at least two steps in their DRSS score [30]. Eyes with severe and very severe NPDR (levels 47–53 in the DRSS) benefited the most. For instance in the RISE and RIDE trials, eyes with severe and very severe NPDR 75% had at least a two-step DRSS improvement [31]. Similarly, following two years of intravitreal aflibercept, 29–37% of aflibercept-treated eyes had at least a two-step improvement in the DRSS compared with 8–16% of macular laser-treated eyes [32]. Protocol T of the DRCR network compared aflibercept, ranibizumab, and bevacizumab in eyes with DME. All of the eyes received 6-monthly injections of the drug and then were reinjected according to need. At 12 months, eyes with DME and NPDR treated with bevacizumab (22%) were less likely to experience an improvement in DRSS compared with eyes treated with aflibercept (31%) or ranibizumab (38%). However, these differences vanished by the second year. In eyes with DME and PDR, aflibercept (76%) was superior to ranibizumab (55%) and bevacizumab (31%) in causing an improvement in the DRSS [29]. Interpreting DRSS improvement in eyes undergoing intravitreal anti-VEGF treatment warrants caution [33]. In a retrospective review of 18 eyes that underwent three consecutive monthly anti-VEGF injections, ultra-wide-field color photos and fluorescein angiograms were compared at baseline and 1 month after the last injection. Despite an improvement by at least one step in the DRSS score in 61% of eyes, the corresponding fluorescein angiograms in these same eyes showed that there was no arteriole or venule reperfusion in the nonperfused areas. Therefore, eyes with DRSS improvement may still be at high risk of developing PDR, particularly if anti-VEGF treatment is suspended [33,34].

Given the improvement in the DRSS in eyes with NPDR/PDR with concurrent DME, the question becomes what role, if any, does VEGF inhibition have in eyes with NPDR in preventing progression to PDR or development of DME. PANORAMA assessed the efficacy and safety of aflibercept for the improvement of severe to moderately severe NPDR by measuring the proportion of eyes that improved at least two steps in the DRSS from the baseline. The study randomized eyes into three arms: sham injections and two different dosing regimens of aflibercept. Patients were followed for 100 weeks [35]. Similarly, the DRCR Protocol W determined whether or not intravitreal aflibercept was able to prevent the development of vision-threatening diabetic retinopathy in high-risk eyes. Eyes were randomized to sham injections or aflibercept injected at baseline, month 1, month 2, month 4, and then every 4 months until year 2 [36]. Chronic frequent treatment with VEGF inhibitors has been shown to improve DR severity and reduce the risk of progression of vision-threatening complications by more than 70% [35,36]. However, in routine clinical practice, the majority of DR patients without vision-threatening complications are not treated with an anti-VEGF due to the unsustainable burden of frequent intravitreal injections and worse outcomes in eyes that had interrupted or reduced treatment compared with those never treated at all [34].

## 2. Gene Therapy

The main goal of gene therapy is to modify a patient’s cellular gene expression by introducing exogenous genetic material (i.e., DNA or RNA) intracellularly to block an unwanted gene expression or to induce the expression of a desirable protein to cure a diseased condition [37]. The presence of the blood–retinal barrier is key in making the eye an ideal target for gene therapy [37,38]. Its presence allows the retina to become an immune-privileged site, making it less likely to elicit an immunological response following the introduction of a foreign material, such as viral DNA. The blood–retinal barrier compartmentalizes the expression of the therapeutic gene to the eye by preventing the leakage of genetic material into the systemic circulation. The compartmentalization of the therapeutic gene to the eye means that smaller doses of genetic material need to be delivered. In addition, since we all have two eyes, one can treat one eye and use the fellow eye as a natural control to assess the efficacy and safety of the treatment. Finally, the eye, because of its transparent media, is directly visualized which allows for precise vector delivery and retinal imaging for monitoring purposes [37].

### 2.1. Gene Therapy as a Biofactory

In 2017, voretigene neparvovec, a recombinant AAV-based gene therapy that replaces the deficient RPE65 gene through delivery to the subretinal space, was approved by several regulatory agencies in the world for the treatment of Leber’s congenital amaurosis [39,40]. This opened the door to the exploration of gene therapy for not only monogenic conditions, such as inherited retinal diseases, but also to other common multifactorial conditions, such as DME and DR. The objective in these cases is to use gene therapy as a biofactory to encode a transgene to express a non-native protein that inhibits VEGF in the target tissue. The first step is to create a transgene and couple it to an appropriate vector. Then, the vector carrying the transgene is delivered to the posterior segment of the eye. The vector then transfects retinal cells and takes over their cellular machinery to start producing and secreting the transgene in enough quantities to produce the desired clinical effect [41].

Genetic therapy may offer a durable and possible curative clinical effect with a single treatment. The continuous release of an anti-VEGF agent could be beneficial in reducing the treatment burden, improving pharmacokinetics, and reducing complications associated with intravitreal injections, leading to an improvement in visual outcomes for patients [42].

### 2.2. Genome Editing 

Gene-editing tools, including zinc finger nucleases and transcription activator-like effector nucleases, have been used to edit DNA. The targeted cleavage of genomic DNA results from the tethering of non-specific nucleases to sequence-specific DNA-binding domains. The limitations of these tools include their high cost and time involved in developing the target sequences. The clustered regularly interspaced short palindromic repeats-associated protein (CRISPR) system may be used as a programmable gene-editing platform. CRISPR has made gene editing much quicker and cheaper [41]. The CRISPR system is an adaptive immune system developed by prokaryotic organisms to fend off viruses. CRISPR recognizes foreign DNA via a single-guide RNA (sgRNA) that is coupled to an endonuclease, such as Cas9. The sgRNA targets a specific sequence in the genome that contains the invading foreign DNA. The endonuclease acts as a molecular scissor that cuts and creates double-strand DNA breaks in this specific genomic sequence. As a result, insertions/deletions (indels) of nucleotides cause frameshift mutations inactivating the gene [41]. 

Endonucleases associated with CRISPR can be programmed using a gRNA to target a specific DNA sequence of interest. The Cas9 from *Streptococcus pyogenes* (SpCas9, 4.1 kb) has been the most used to date. Other orthologs from *Staphylococcus aureus* (Sa Cas9, 3.1 kb) and *Campylobacter jejuni* (Cj Cas 9, 3 kb) have also been studied [42]. In the cases of DR and DME, there is no specific pathogenic mutation of a deficient gene. Therefore the objective would be to target and inactivate genes involved with the VEGF pathway, such as the *VEGF-A, VEGFR-2*, and *HIF-1* alpha genes by using CRISPR/Cas9 [42,43,44,45,46,47,48]. 

There are several limitations to CRISPR. Even under perfect conditions, CRISPR is not very efficient, leading to a variable DNA cleavage frequency. Some of the induced indels could remain silent. In addition, double-stranded DNA breaks on both alleles are not always induced as planned. The size of the most widely used Cas9, SpCas9, is too large to be packaged with its sgRNA in a single AAV vector. So, in order to use SpCas9 a dual-vector system is required. Dual vectors are less efficient for infection than single vectors [41]. Finally, off-target mutations induced by CRISPR-Cas systems are of concern [41,49]. The specificity of CRISPR-based therapies is crucial, requiring targeting of the correct cell types for effective treatment. To minimize off-target effects, strategies such as truncated gRNAs, high-fidelity Cas9 variants, anti-CRISPR proteins, and self-destructing CRISPR systems are being developed. Ultimately, improving cell targeting and minimizing unintended mutations will be key to advancing CRISPR-based treatments for ocular diseases [50].

### 2.3. Vectors

In order for therapeutic gene products to be delivered to target cells, the use of a gene-delivery system is required. These carrier molecules are called vectors. The role of vectors is to safely and effectively deliver therapeutic genes to target cells by overcoming intra and extracellular barriers innate to living organisms against foreign genetic material, facilitating transport into the nuclear compartment of target cells without evoking an immune response that would result in unwanted side effects. Vectors can be viral or nonviral. The ideal vector should lead to sustained levels of gene expression, large capacity, low toxicity, low risk of mutations, and low immunogenicity [51].

#### 2.3.1. Non-Viral Vectors

Non-viral vectors have been recently emerging as alternatives to viral vectors. The main problems posed by viral vectors are immunogenicity and cytotoxicity, as well as potential of insertional mutagenesis [52].The advantages of nonviral vectors include a high loading capacity, enabling the possibility for the simultaneous delivery of multiple therapeutic genes, low risk of immunogenicity and insertional mutations, and a less expensive manufacturing process [53]. Nonviral vectors use either physical or chemical systems to transport DNA or RNA across membranes. Examples of non-viral vectors include polymers, liposomes, nanoparticles, aptamers, antisense oligonucleotides, and small interfering RNA. In spite of being a promising alternative to viral vectors, nonviral vectors continue to be less efficient in delivering the therapeutic gene to target cells [54].

#### 2.3.2. Viral Vectors

Because of their biological properties, viruses serve as the natural choice for vectors in gene therapy. The effectiveness of transduction and transgenic expression depends on several factors, including the capsid, promoter, nature of the transgene, other cis-regulatory factors, and the delivery approach. Several viral vectors are currently used for retinal gene therapy, primarily adeno-associated virus (AAV) and lentiviral vectors [55,56,57,58,59,60,61].

- Adenovirus

Adenoviruses are characterized by a double-stranded DNA and a non-enveloped icosahedral structure, which includes three viral capsid proteins. Its genome consists of an early region with four transcription units (E1–E4), and a late region with five transcription units (L1–L5). Several adenoviral subtypes have been shown to have low virulence and high transduction efficiency. These adenoviral vectors can also be produced in high quantities. They pose a low risk of insertional mutagenesis because of their inability to integrate into the host’s genome [62]. However, their use is limited by several factors, such as transient transgene expression by the AdV vector, which leads to a subtherapeutic effect. They have also been known to activate innate immunity, leading to severe toxicity in high vector dosages. Lastly, around 80% of the human population is estimated to have been exposed to adenovirus, which results in the presence of “pre-existing vector immunity”, which means a higher vector dose is required to achieve a therapeutic effect. For these reasons, adenovirus vectors have fallen out of use and other viral vectors are currently preferred for ocular gene therapy [63].

b Adeno-Associated Virus (AAV)

AAV is a smaller, non-enveloped single-stranded DNA virus, a member of the parvovirus family that depends on other viruses to replicate. The virus contains two genes, *rep* and *cap*, that encode polypeptides for viral packing and replication. Recombinant AAV (AAVr) can be designed by excising the *rep* and *cap* genes from the viral genome and inserting therapeutic genes in their place. The advantages of AAVr include their non-pathogenicity, the need for a helper virus to replicate, low immunogenicity, the ability to transduce non-dividing neuronal cells, such as photoreceptors and the RPE, lasting and stable transgene expression, and the inability to integrate into the host’s genome. Because of these characteristics, it is currently a leading vector for retinal gene therapy. The main disadvantages of AAVr as a viral vector are its limited capacity to carry up to 5 kb of genetic material and the pre-existence or development of anti-AAV-neutralizing antibodies which may limit its transduction efficacy [64,65].

c Lentivirus

Lentiviruses form part of the retroviradae family. The genome consists of a capsid that envelops a single strand of RNA of 8 to 10 kb, an integrase, a reverse-transcriptase, and a protease. There are several advantages of utilizing a lentivirus as a vector. Lentiviruses are able to infect non-dividing cells, like photoreceptors, integrate into the host’s genome, carry a larger genetic load, exhibit broad tropism, and elicit a lower immune response [66]. However, in the particular case of DR or DME, lentiviruses do not offer additional advantages over AAV, since there is no need to carry a larger genetic load.

The main disadvantage of a lentivirus vector is the potential risk of insertional mutagenesis if it integrates itself into a tumor-suppressor gene. Because the eye is isolated from the systemic circulation by the blood–retina barrier, there is a lower risk with ocular targets compared with systemic targets. In addition, to improve the safety of lentivirus as a vector, two approaches have been used. First, the removal of any non-essential viral sequences from the vector is mandated. Second, self-inactivating sequences that significantly reduce the risk of insertional mutagenesis can be engineered into the vector [67,68,69].

#### 2.3.3. Routes of Administration for Retinal Vector Delivery

The route of administration is a major determinant of the efficacy of gene therapy. Subretinal, intravitreal, and suprachoroidal injections are currently being explored for retinal gene therapy. Each one of these has advantages and disadvantages.

Subretinal injections require pars plana vitrectomy (PPV) followed by a small retinotomy in order to administer the viral vectors. PPVs are generally performed in the operating room and may require general anesthesia. This may limit widespread accessibility to all patients in need of such therapy. Injection into the subretinal space will lead to a temporary retinal detachment that allows for a direct delivery to the retinal pigment epithelium (RPE) and photoreceptors of the vector. The subretinal space is an immune-privileged site which minimizes the risk of the host eliciting an immune response to the viral capsid antigens. Subretinal injections oflentiviral or AAVr vectors transduce the RPE and the photoreceptors in an efficient and stable manner, but are generally limited to the area where the bleb was created [70,71,72,73,74,75]. Potential injury to the photoreceptors due toiatrogenic retinal detachment may occur and care must be taken to avoid creating an iatrogenic macular hole [76,77]. Subretinal injections may be associated with varying degrees of reflux of the genetic vector into the vitreous cavity.

Most ophthalmologists are very familiar with intravitreal injections. Intravitreal injections have a safer profile in comparison with the subretinal method. They have fewer complications associated with the procedure of administration and can be performed with topical anesthesia in the office setting. An intravitreal delivery route is better able to target inner retinal cells, such as ganglion, bipolar, and Müller cells. However, the vector delivered intravitreally needs to overcome several immunological and physical barriers before the genetic vector can successfully transduce the target cells. A higher dose of the vector needs to be injected to compensate for the dilution effect that occurs once the vectoris injected intravitreally. A stronger immune response may be elicited after a higher dose. If the host has pre-existing antibodies against the viral vector, neutralization of the vector may trigger a stronger immune response from the intravitreal injection. The vector needs to navigate long diffusion distances across the vitreous cavity to reach the target cells in the retina. In the case of DR, where the purpose of gene therapy is to infect cells to convert them into an anti-VEGF biofactory, precise transduction of RPE and photoreceptor cells is not that important, unlike in eyes with inherited retinal diseases. Another disadvantage is the possibility of the off-target transduction of anterior segment structures, like the ciliary body, iris, and corneal endothelium. The internal limiting membrane (ILM), which acts as a physical and biological barrier, needs to be breached before the genetic material reaches the retinal cells This is an important consideration if the goal is to transfect the RPE or photoreceptors, which is not the case in DME or DR. Heparan sulfate proteoglycan, which contains binding sites for both AAV2 and AAV3, is abundantly present in the ILM. These binding sites allow for a buildup of viral particles at the vitreoretinal interface, limiting diffusion through the ILM [78]. Directed evolution is one way of overcoming the ILM barrier to transduce outer retinal neurons following an intravitreal injection. In directed evolution, vector variant libraries are rapidly screened for cell tropism and transduction efficiency. The AAV2-7m8 vector is an example of a vector developed by directed evolution [79]. Finally, once the vector and its cargo reach the intended retinal cells, the capsid may be ubiquinated, which leads to proteasome degradation and less efficient transduction of the genetic material [78].

A suprachoroidal injection is an office-based procedure that is easier to perform and safer when compared with a subretinal injection. There is a potential space along the inner surface of the sclera that separates the choroid from the sclera upon expansion with fluid [80]. Specialized microneedles are needed to access the suprachoroidal space. Suprachoroidal delivery of drugs or gene therapy is an attractive delivery route, since it can potentially cover a greater surface area when compared with a subretinal injection, which is limited by the bleb created during the subretinal injection of the vector. Some AAV vectors may have difficulties in crossing the choroid in their attempt to reach the RPE and photoreceptors [81]. Other disadvantages include the theoretical high likelihood of eliciting a systemic immune response, since the vector is being injected into a highly vascularized area. The results of the ALTITUDE study, where higher doses of the RGX-314 were injected into the suprachoroidal space without eliciting a higher inflammatory response, suggest otherwise. Both cellular and humoral immune responses are elicited against the transgene protein [82].

## 3. Genetic Therapy Targeting the VEGF Pathway for Diabetic Retinopathy or Diabetic Macular Edema

The main strategy of gene therapy for DR has focused on delivering transgenes that express anti-angiogenic proteins that directly or indirectly inhibit the VEGF pathway. Table 1 summarizes all the human trials of different transgenes studied in NPDR and DME.

### 3.1. RGX-314 (ABBV-RGX-314) (Rockville, MD, USA)

RGX-314 is a rAAV8 vector that expresses a ranibizumab-like anti-VEGF soluble monoclonal antibody fragment. AAV8 is capable of transducing both RPE and photoreceptor cells after a subretinal injection of a low to moderate dose, leading to the expression of an anti-human VEGF antibody fragment [83].

ALTITUDE is a phase-2 multi-center, open-label, randomized, controlled, dose-escalation trial. The primary outcome was the proportion of eyes with ≥two-step improvement in the DRSS at one year. Secondary outcomes included the need for additional standard of care interventions, development of DR-related ocular complications, and the efficacy, safety, and tolerability of ABBV-RGX-314 in patients with moderately severe or severe NPDR or mild PDR with no active center-involved DME (CST < 320 µm). BCVA had to be ≥20/40 and no eye was allowed if they had received an anti-VEGF injection in the prior 6 months. ABBV-RGX-314 was delivered to the suprachoroidal space via a SCS Microinjector^TM^ in an office setting. Three dose levels (2.5 × 10^11^, 5 × 10^11^, and 1 × 10^12^ vg/eye respectively) are being evaluated. No prophylactic corticosteroids were administered in doses 1 and 2 [84].

The interim 12-month results of dose level 1 (n = 15) and 2 (n = 35) demonstrated that there was a clinically meaningful improvement in disease severity and reduction of vision-threatening events, particularly in NPDR eyes. When only NPDR eyes were considered, the control eyes (n = 8) demonstrated that 37.5% of eyes had a ≥two-step worsening in the DRSS and only 12.5% of eyes had an improvement of two steps in the DRSS. In contrast, dose level 1 showed ≥two-step worsening in 16.7% compared with 33.3% two-step improvement. Dose level 2 prevented disease progression in all NPDR patients with 0% worsening of two steps in the DRSS compared with 20.8% two-step improvement in the DRSS. Vision-threatening events were observed in 37.5%, 16.7%, and 4.2% in the control, dose level 1, and dose level 2 groups, respectively. Dose level 2 reduced vision-threatening events by 89%. Suprachoroidal ABBV-RGX-314 was well-tolerated in dose levels 1 and 2 through 12 months. Episcleritis was seen in 6.7% to 14.3% of eyes, depending on the dose level. All cases were mild to moderate and resolved with topical corticosteroids. Intraocular inflammation was only observed in dose level 2. It affected 8.6% of eyes and was considered to be mild in all cases. Most presented 2–6 weeks post-injection as anterior cells in slit lamp examination. All cases resolved with topical corticosteroids [84].

### 3.2. 4D-150 (4D Molecular Therapeutics, Emeryville, CA, USA)

4D-150 consists of a non-human primate-evolved R100 capsid carrying a transgene payload that consists of aflibercept and VEGF-C RNAi that inhibits VEGF-A, VEGF-B, VEGF-C, and PlGF. There is a robust pan-retinal transgene expression causing the simultaneous inhibition of VEGF-A, VEGF-B, VEGF-C, and PlGF following a single intravitreal injection [85].

SPECTRA is a phase-2 multicenter, randomized, double-masked study designed to assess the safety, efficacy, and determination of the best dose of a single intravitreal 4D-150 injection in treatment-naive and previously treated DME eyes. Twenty-two eyes were randomized to 5 × 10^9^ vg/eye (n = 1), 1 × 10^10^ vg/eye (n = 12), or 3 × 10^10^ vg/eye (n = 9) of 4D-150. Patients were to be followed monthly for 104 weeks. Prior to the intravitreal injection of 4D-150, all eyes received three consecutive monthly intravitreal injections of aflibercept 2 mg. The first aflibercept was administered 8 weeks prior to 4D-150 injection. The third aflibercept was injected 2 weeks after 4D-150 injection. The eyes started a prophylactic difluprednate topical 16-week taper three days prior to the intravitreal 4D-150 injection. Inclusion criteria included DME diagnosis within the past 2 years, a central subfield thickness (CST) of ≥350 µm, and demonstration of responsiveness to VEGF inhibition. Responsiveness to VEGF inhibition was assessed by observing a decrease of ≥40 µm in the CST at 1 week prior to 4D-150 injection when compared with the baseline. Eight weeks after 4D-150 injection, eyes were assessed for the need for supplemental aflibercept 2 mg. Eyes received supplemental aflibercept if CST had an increase of ≥50 µm. Eyes continued to receive supplemental aflibercept until the change in CST was ≤30 µm on two consecutive visits or the CST was ≤325 µm [86].

The interim results of SPECTRA revealed that 4D-150 demonstrated a sustained improvement and dose response in visual acuity and CST through 32 weeks. It was well-tolerated, with no intraocular inflammation observed at any timepoint or dose level. The 3 × 10^10^ vg/eye was chosen as the Phase-3 dose. Nine eyes received this dose and had a mean gain in BCVA of 8.4 letters and a mean reduction in the central subfield thickness of 194 µm. There was an 86% reduction in treatment burden when compared with projected on-label aflibercept 2 mg administered every 8 weeks. Furthermore, 89% of eyes required 0–1 supplemental injections of aflibercept and 56% of eyes did not require a single supplemental injection of aflibercept. All patients completed the 16-week topical steroid taper on schedule and all patients remained completely off steroids. There was no hypotony, endophthalmitis, vasculitis, choroidal effusions, or retinal artery occlusions [86].

### 3.3. Ixoberogene Soroparvovec (Ixo-Vec, ADVM-022) (Adverum Biotechnologies, Redwood Cities, CA, USA)

Ixoberogene soroparvovec uses the recombinant adeno-associated virus capsid AAV2.7m8, which was derived from AAV2 through directed evolution to enhance efficacious retinal cell transduction following an intravitreal injection [79]. Ixo-vec delivers a coding sequence which allows for the continuous expression of an aflibercept therapeutic dose [87].

INFINITY was a phase-2 trial designed to compare standard of care intravitreal aflibercept 2 mg (n = 9) with a single intravitreal injection of Ixo-vec of either 2 × 10^11^ vg/eye (n = 13) or 6 × 10^11^ vg/eye (n = 12) in DME eyes. Thirty-six patients with recently diagnosed DME were enrolled in the study. The patients were evaluated monthly for 48 months and profilactically treated with topical difluprednate for 10 weeks. All three groups received an intravitreal injection of aflibercept 2 mg at baseline. The ixo-vec groups received their injection on day 8. Starting at week 8, supplemental aflibercept injections were administered if the CST ≥ 50 µm when compared with that on day 1 and week 4, or there was a loss of >five letters of BCVA when compared with the higher of two measurements recorded on day 1 or week 4. The primary endpoint of the study was the time to the first supplemental aflibercept injection [88].

After a median follow-up of 30 weeks, 89% of the control aflibercept 2 mg group required supplemental aflibercept. This contrasts with the 39% of the low-dose and 25% of the high-dose Ixo-vec groups that required aflibercept rescue treatment. The BCVA was maintained in all groups until week 24. Thereafter, the high-dose Ixo-vec group experienced loss of vision secondary to the development of adverse events. The CST results were also maintained through 34 weeks. At 24 weeks, 29% of the aflibercept control group demonstrated a ≥two-step improvement in the DRSS compared with 46% in both the low and high doses of Ixo-vec.

Dose-dependent inflammation was observed, with a dose-limiting toxicity of 6 × 10^11^ vg/eye. Almost all patients treated with Ixo-vec developed intraocular inflammation. Most of these inflammatory events were in the anterior segment of the eye (transilluminations defects of the iris, synechiae, and pigmentary changes of the iris). Posterior segment inflammation was observed in 8–17% of eyes, depending on the dose. The study was unmasked in April of 2021 because a patient in the higher-dose group developed severe hypotony. Two more patients eventually also developed hypotony. These three eyes developed serous choroidal detachments, panuveitis, and corneal decompensation. All three eyes required surgical intervention and received a fluocinolone acetonide intravitreal implant. All the eyes in the 6 × 10^11^ vg/eye dose group required additional anti-inflammatory treatment beyond difluprednate. No hypotony was observed in the 2 × 10^11^ vg/eye dose group. There has been speculation that the comorbid nature of the study population was partly responsible for the complications observed in the higher dose. Remarkably, this same dose was used in a cohort of NV-AMD patients with no hypotony observed. These results led Adverum to discontinue their DME program and only concentrate on their NV-AMD program [88].

### 3.4. Potential Risks of Gene Therapy

Dreaded potential risks of gene therapy include death and oncogenesis [89,90,91,92]. In 2009, eighteen hours after a hepatic artery infusion of a recombinant human adenovirus type-5 carrying the ornithine transcarbamylase cDNA at a dose of 6 × 10^11^ particles/kg, an 18 year old patient with partial ornithine transcarbamylase deficiency developed systemic inflammatory response syndrome, which led to multi-organ failure and his demise [90].

Malignant transformation via insertational mutagenesis and integration of the viral DNA inserts into the host’s DNA through accidental activation of oncogenes or inactivation of tumor-suppressor genes have also been documented [93,94,95]. Nine infants with x-linked severe combined immunodeficiency were essentially cured following a retrovirus-mediated ex vivo gene transfer that reconstituted their immune system. Unfortunately, three years after gene therapy, two patients developed a T cell leukemia. In both of these cases, the leukemia was induced by an unintended activation of LMO2 when the retroviral vector inserted itself at or near the *LMO2* gene, which has been linked to leukemia [93,94,95].

As the INFINITY trial demonstrated, severe inflammatory reactions may occur following ocular gene therapy and lead to irreversible ocular structural damage. In up to 50% of patients receiving ocular gene therapy, an adverse ocular inflammatory reaction has been reported and named gene therapy-associated uveitis (GTAU). The clinical presentation of GTAU varies among patients and appears to depend on the route of delivery. Almost 50% of eyes receiving an intravitreal injection of a genetic vector develop GTAU compared with 28% after a subretinal injection and 21% after a suprachoroidal injection [96]. Vitritis and anterior segment inflammation are the most common manifestations of GTAU following an intravitreal injection of a genetic vector. Chorioretinitis, choroidal thickening, and subretinal deposits are commonly seen after a subretinal injection. A dose-dependent episcleritis has been reported in several patients after a suprachoroidal injection [96]. In addition, an immune response may be elicited against any vector component or transgene product. Impurities from the manufacturing process may contaminate the formulation and give rise to immune responses. Host-related factors, such as gender, age, and the underlying retinal disease may determine the risk of GTAU [96].

## 4. Conclusions and Future Directions

Unfortunately, the current diabetes epidemic does not have an end in sight. Future projections are staggering and, despite advances in the treatment of vision-threatening complications of DR, such as DME and PDR, the cases continue to increase year after year. Healthcare systems are being stretched thin by the high treatment burden of DR. There are limited resources and a high volume of patients. The costs, patient fatigue, and a perception of treatment failure negatively affect adherence to treatment. A one-time in-office injection of gene therapy may potentially provide a long-lasting improvement in DR severity and reduce the risk of vision-threatening complications in eyes with NPDR. In eyes with DME, gene therapy may decrease the burden of treatment and close the gap between clinical trial outcomes and routine clinical practice.

Current ocular gene therapy for DR and DME is based on the concept of creating a biofactory that produces an anti-VEGF protein in a sustained manner. Despite promising results in the ALTITUDE and SPECTRA trials, one must remain vigilant for adverse events. As the results of the INFINITY trial demonstrated, severe inflammatory reactions may occur and lead to irreversible ocular structural damage [88]. Frequent monitoring visits for inflammation may end up being as burdensome as current anti-VEGF visits. In order to avoid these complications, GTAU needs to be minimized. However our current understanding of the underlying immune response remains incomplete and the best strategies for prophylaxis, treatment, and monitoring of GTAU remain unclear [96]. Future research directions need to include standardizing vector characterization, refining animal models, and identifying biomarkers to detect and quantify immune responses within the retina [97]. There is a theoretical concern that chronic VEGF suppression may be detrimental to ocular health and with gene therapy, there is no way to turn it off. In the normal healthy eye, the RPE secretes VEGF that is essential for a healthy choriocapillaris. The choriocapillaris endothelial cell fenestrations in non-human primates were significantly reduced following VEGF inhibition by bevacizumab [98]. These findings have not been reported in humans, to the best of our knowledge.

Finally, the costs of gene therapy need to be taken into account. For instance, voretigene neparvovec with a price tag of USD 850,000 per patient is not a cheap treatment. The economics of a rare inherited disease like *RPE65* Leber’s congenital amaurosis are very different from a common condition, such as DME or DR. In the event that gene therapy becomes a reality for DME and DR, we hope that it is priced affordably for most of the population at risk.

## Figures and Tables

**Table 1:** Summary of ocular gene therapy trials idiabetic retinopathy and diabetic macular edema.

	RGX-314	ADV-022 Ixoberogene Ixo-Vec	4D-150
Sponsor	Regenxbio—Abbie	Adverum	Molecular Therapeutics
Transgene Product	Ranibizumab-like anti-VEGF monoclonal antibody fragment	Aflibercept-like	Aflibercept-like plus VEGF-C inhibitory RNA
Vector	AAV8	AAV2.7m8	AAV-R100
Clinical Trial	ALTITUDE	INFINITY	SPECTRA
Route of Delivery	Suprachoroidal	Intravitreal	Intravitreal
Dose (vg/eye)	2.5 × 10^11^ (N = 15) 5 × 10^11^ (N= 35) 1 × 10^12^	2 × 10^11^ (N = 12) 6 × 10^11^ (N = 13)	5 × 10^9^ (N = 1) 1 × 10^10^ (N = 11) 3 × 10^10^ (N = 9)
Therapeutic Indication	NPDR	DME	DME
Anti-Inflammatory Prophylaxis	Low Dose = None Middle Dose = None High Dose = Topical Steroids	Topical Difluprednate 0.05% for 10 wks	Topical Difluprednate 0.05% for 16 wks
BCVA Outcomes (Letters)	Not Reported	AFL control = +7.5 Low dose = +8.8 High dose = −6.9	Middle Dose = +8.4 High Dose = +7.1
Central Subfield Thickness Outcomes (µm)	Not Reported	AFL control = −117 Low dose = −152 High dose = −144	Middle Dose =−194 Low Dose = −153
Rescue anti-VEGF Requirement		AFL control = 89% Low Dose = 39% High Dose= 25%	
Treatment Burden Reduction			Middle Dose = 65% High Dose = 86%
DRSS ≥ 2 Step Improvement	Control = 12.5% Low Dose = 33.3% Middle Dose = 20.8%	AFL control = 29% Low dose = 46% High dose = 46%	NOT REPORTED
Development of Vision-Threatening Events (DME and PDR)	Control = 37.5% Low Dose = 16.7% Middle Dose = 4.2%	NOT APPLICABLE	NOT APPLICABLE
Adverse Events Intraocular Inflammation	Low Dose = 0% Middle Dose = 8.6%	AFL control = 33% Low dose = 92% High dose = 83%	Middle Dose = 0% Low Dose = 0%
Adverse Events Episcleritis	Low Dose = 6.7% Middle Dose = 14.3%	AFL control = 0% Low dose = 0% High dose = 0%	Middle dose = 0% High dose = 0%
Adverse Events Increase Intraocular Pressure	Low Dose = 6.7% Middle Dose = 8.6%	AFL Control = 0% Low dose = 0% High dose = 0%	Middle dose = 0% High dose = 0%
Adverse Events Hypotony	Low Dose = 0% Middle Dose = 0%	AFL control = 0% Low dose = 0% High dose = 25%	Middle dose = 0% High dose = 0%

## References

[1] IDF Diabetes Atlas. https://diabetesatlas.org.

[2] Klein R., Klein B.E., Moss S.E. (1984). Visual impairment in diabetes. Ophthalmology.

[3] Williams R., Airey M., Baxter H., Forrester J., Kennedy-Martin T., Girach A. (2004). Epidemiology of diabetic retinopathy and macular oedema: A systematic review. Eye.

[4] Brown M.M., Brown G.C., Sharma S., Shah G. (1999). Utility values and diabetic retinopathy. Am. J. Ophthalmol..

[5] Javitt J.C., Aiello L.P., Chiang Y., Ferris F.L., Canner J.K., Greenfield S. (1994). Preventive eye care in people with diabetes is cost-saving to the federal government. Implications for health-care reform. Diabetes Care.

[6] Javitt J.C., Aiello L.P. (1996). Cost-effectiveness of detecting and treating diabetic retinopathy. Ann. Intern. Med..

[7] Tan T.E., Wong T.Y. (2022). Diabetic retinopathy: Looking forward to 2030. Front. Endocrinol..

[8] Engerman R.L., Kern T.S. (1995). Retinopathy in animal models of diabetes. Diabetes Metab. Rev..

[9] Klein R., Klein B.E., Moss S.E., Cruickshanks K.J. (1994). The Wisconsin Epidemiologic Study of diabetic retinopathy. XIV. Ten-year incidence and progression of diabetic retinopathy. Arch. Ophthalmol..

[10] Singh A., Stewart J.M. (2009). Pathophysiology of diabetic macular edema. Int. Ophthalmol. Clin..

[11] Wu L., Acon D., Wu A., Wu M. (2019). Vascular endothelial growth factor inhibition and proliferative diabetic retinopathy, a changing treatment paradigm?. Taiwan J. Ophthalmol..

[12] Li H.Y., Yuan Y., Fu Y.H., Wang Y., Gao X.Y. (2020). Hypoxia-inducible factor-1alpha: A promising therapeutic target for vasculopathy in diabetic retinopathy. Pharmacol. Res..

[13] Ferrara N. (2004). Vascular endothelial growth factor: Basic science and clinical progress. Endocr. Rev..

[14] Koch S., Claesson-Welsh L. (2012). Signal transduction by vascular endothelial growth factor receptors. Cold Spring Harb. Perspect. Med..

[15] Diabetic Retinopathy Study (1981). Report Number 6. Design, methods, and baseline results. Report Number 7. A modification of the Airlie House classification of diabetic retinopathy. Prepared by the Diabetic Retinopathy. Invest. Ophthalmol. Vis. Sci..

[16] Grading diabetic retinopathy from stereoscopic color fundus photographs—An extension of the modified Airlie House classification (1991). ETDRS report number 10. Early Treatment Diabetic Retinopathy Study Research Group. Ophthalmology.

[17] Klein R., Klein B.E., Moss S.E. (2001). How many steps of progression of diabetic retinopathy are meaningful? The Wisconsin epidemiologic study of diabetic retinopathy. Arch. Ophthalmol..

[18] Zas M., Cotic M., Wu M., Wu A., Wu L. (2020). Macular laser photocoagulation in the management of diabetic macular edema: Still relevant in 2020?. Taiwan J. Ophthalmol..

[19] Virgili G., Curran K., Lucenteforte E., Peto T., Parravano M. (2023). Anti-vascular endothelial growth factor for diabetic macular oedema: A network meta-analysis. Cochrane Database Syst. Rev..

[20] Gross J.G., Glassman A.R., Jampol L.M., Inusah S., Aiello L.P., Antoszyk A.N., Baker C.W., Berger B.B., Bressler N.M., Writing Committee for the Diabetic Retinopathy Clinical Research Network (2015). Panretinal Photocoagulation vs Intravitreous Ranibizumab for Proliferative Diabetic Retinopathy: A Randomized Clinical Trial. JAMA.

[21] Gross J.G., Glassman A.R., Liu D., Sun J.K., Antoszyk A.N., Baker C.W., Bressler N.M., Elman M.J., Ferris F.L., Gardner T.W. (2018). Five-Year Outcomes of Panretinal Photocoagulation vs Intravitreous Ranibizumab for Proliferative Diabetic Retinopathy: A Randomized Clinical Trial. JAMA Ophthalmol..

[22] Beaulieu W.T., Bressler N.M., Melia M., Owsley C., Mein C.E., Gross J.C., Jampol L.M., Glassman A.R. (2016). Panretinal Photocoagulation Versus Ranibizumab for Proliferative Diabetic Retinopathy: Patient-Centered Outcomes From a Randomized Clinical Trial. Am. J. Ophthalmol..

[23] Sivaprasad S., Prevost A.T., Vasconcelos J.C., Ridell A., Murphy C., Kelly J., Bainbridge J., Tudor-Edwards R., Hopkins D., Hykin P. (2017). Clinical efficacy of intravitreal aflibercept versus panretinal photocoagulation for best corrected visual acuity in patients with proliferative diabetic retinopathy at 52 weeks (CLARITY): A multicentre, single-blinded, randomised, controlled, phase 2b, non-inferiority trial. Lancet.

[24] Obeid A., Gao X., Ali F.S., Talcott K.E., Aderman C.M., Hyman L., Ho A.C., Hsu J. (2018). Loss to Follow-Up in Patients with Proliferative Diabetic Retinopathy after Panretinal Photocoagulation or Intravitreal Anti-VEGF Injections. Ophthalmology.

[25] Obeid A., Su D., Patel S.N., Uhr J.H., Borkar D., Gao X., Fineman M.S., Regilio C.D., Maguire J.I., Garg S.J. (2019). Outcomes of Eyes Lost to Follow-up with Proliferative Diabetic Retinopathy That Received Panretinal Photocoagulation versus Intravitreal Anti-Vascular Endothelial Growth Factor. Ophthalmology.

[26] Wubben T.J., Johnson M.W., Anti VTISG (2019). Anti-VEGF Therapy for Diabetic Retinopathy: Consequences of Inadvertent Treatment Interruptions. Am. J. Ophthalmol..

[27] Bressler S.B., Liu D., Glassman A.R., Blodi B.A., Castellarin A.A., Jampol L.M., Kaufman P.L., Melia M., Singh H., Wells J.A. (2017). Change in Diabetic Retinopathy Through 2 Years: Secondary Analysis of a Randomized Clinical Trial Comparing Aflibercept, Bevacizumab, and Ranibizumab. JAMA Ophthalmol..

[28] Ip M.S., Domalpally A., Hopkins J.J., Wong P., Ehrlich J.S. (2012). Long-term effects of ranibizumab on diabetic retinopathy severity and progression. Arch. Ophthalmol..

[29] Wykoff C.C. (2017). Impact of intravitreal pharmacotherapies including antivascular endothelial growth factor and corticosteroid agents on diabetic retinopathy. Curr. Opin. Ophthalmol..

[30] Brown D.M., Schmidt-Erfurth U., Do D.V., Holz F.G., Boyer D.S., Midena E., Heier J.S., Terasaki H., Kaiser P.K., Marcus D.M. (2015). Intravitreal Aflibercept for Diabetic Macular Edema: 100-Week Results From the VISTA and VIVID Studies. Ophthalmology.

[31] Bonnin S., Dupas B., Lavia C., Erginay A., Dhundass M., Couturier A., Gaudric A., Tadayoni R. (2019). Anti-Vascular Endothelial Growth Factor Therapy Can Improve Diabetic Retinopathy Score without Change in Retinal Perfusion. Retina.

[32] Goldberg R.A., Hill L., Davis T., Stoilov I. (2022). Effect of less aggressive treatment on diabetic retinopathy severity scale scores: Analyses of the RIDE and RISE open-label extension. BMJ Open Ophthalmol..

[33] Brown D.M., Wykoff C.C., Boyer D., Heir S.D., Clark W.L., Emanuelli A., Higgins P.M., Singer M., Weinreich D.M., Yancopoulos G.D. (2021). Evaluation of Intravitreal Aflibercept for the Treatment of Severe Nonproliferative Diabetic Retinopathy: Results From the PANORAMA Randomized Clinical Trial. JAMA Ophthalmol..

[34] Maturi R.K., Glassman A.R., Josic K., Baker C.W., Gerstenblith A.T., Jampol L.M., Meleth A., Martin D.F., Melia M., Punjabi O.S. (2023). Four-Year Visual Outcomes in the Protocol W Randomized Trial of Intravitreous Aflibercept for Prevention of Vision-Threatening Complications of Diabetic Retinopathy. JAMA.

[35] Amato A., Arrigo A., Aragona E., Manitto M.P., Saladino A., Bandello F., Battaglia Parodi M. (2021). Gene Therapy in Inherited Retinal Diseases: An Update on Current State of the Art. Front. Med..

[36] Kumaran N., Michaelides M., Smith A.J., Ali R.R., Bainbridge J.W.B. (2018). Retinal gene therapy. Br. Med. Bull..

[37] Russell S., Bennett J., Wellman J.A., Chung D.C., Yu Z.F., Tillman A., Wittes J., Pappas J., Elci O., McCague S. (2017). Efficacy and safety of voretigene neparvovec (AAV2-hRPE65v2) in patients with RPE65-mediated inherited retinal dystrophy: A randomised, controlled, open-label, phase 3 trial. Lancet.

[38] Maguire A.M., Russell S., Wellman J.A., Chung D.C., Yu Z., Tillman A., Wittes J., Pappas J., Elci O., Marshall K.A. (2019). Efficacy, Safety, and Durability of Voretigene Neparvovec-rzyl in RPE65 Mutation-Associated Inherited Retinal Dystrophy: Results of Phase 1 and 3 Trials. Ophthalmology.

[39] Sharma A., Wu L., Bloom S., Stanga P., Sosa Lockward J., Weng C.Y., Abbas M.A., Rezaei K.A. (2023). RWC Update: Enhanced ILM Peeling; Retinal Gene Therapy; Laser-Induced Retinal Break and Vitreous Hemorrhage. Ophthalmic Surg. Lasers Imaging Retin..

[40] Lin F.L., Wang P.Y., Chuang Y.F., Wang J.H., Wong V.H.Y., Bui B.V., Liu G.S. (2020). Gene Therapy Intervention in Neovascular Eye Disease: A Recent Update. Mol. Ther..

[41] Yiu G. (2018). Genome Editing in Retinal Diseases using CRISPR Technology. Ophthalmol. Retina.

[42] Kim E., Koo T., Park S.W., Kim D., Kim K., Cho H.Y., Song D.W., Lee K.J., Jung M.H., Kim S. (2017). In vivo genome editing with a small Cas9 orthologue derived from Campylobacter jejuni. Nat. Commun..

[43] Yiu G., Tieu E., Nguyen A.T., Wong B., Smit-McBride Z. (2016). Genomic Disruption of VEGF-A Expression in Human Retinal Pigment Epithelial Cells Using CRISPR-Cas9 Endonuclease. Invest. Ophthalmol. Vis. Sci..

[44] Park J., Cui G., Lee H., Jeong H., Kwak J.J., Lee J., Byeon S.H. (2023). CRISPR/Cas9 mediated specific ablation of vegfa in retinal pigment epithelium efficiently regresses choroidal neovascularization. Sci. Rep..

[45] Huang X., Zhou G., Wu W., Duan Y., Ma G., Song J., Xiao R., Vandenberghe L., Zhang F., D’Amore P.A. (2017). Genome editing abrogates angiogenesis in vivo. Nat. Commun..

[46] Wu W., Duan Y., Ma G., Zhou G., Park-Windhol C., D’Amore P.A., Lei H. (2017). AAV-CRISPR/Cas9-Mediated Depletion of VEGFR2 Blocks Angiogenesis In Vitro. Invest. Ophthalmol. Vis. Sci..

[47] Huang X., Zhou G., Wu W., Ma G., D´Amore P.A., Mukai S., Lei H. (2017). Editing VEGFR2 Blocks VEGF-Induced Activation of Akt and Tube Formation. Invest. Ophthalmol. Vis. Sci..

[48] Koo T., Park S.W., Jo D.H., Kim D., Kim J.H., Cho H.Y., Kim J., Kim J.H., Kim J.S. (2018). CRISPR-LbCpf1 prevents choroidal neovascularization in a mouse model of age-related macular degeneration. Nat. Commun..

[49] Cho G.Y., Shaefer K.A., Bassuk A.G., Tsang S.H., Mahajan V.B. (2018). Crispr Genome Surgery in the Retina in Light of Off-Targeting. Retina.

[50] Chung S.H., Sin T.N., Ngo T., Yiu G. (2020). CRISPR Technology for Ocular Angiogenesis. Front. Genome Ed..

[51] Klink D., Schindelhauer D., Laner A., Tucker T., Bebok Z., Schwiebert E.M., Boyd A.C., Scholte B.J. (2004). Gene delivery systems--gene therapy vectors for cystic fibrosis. J. Cyst. Fibros..

[52] Butt M.H., Zaman M., Ahmad A., Khan R., Mallhi T.H., Hasan M.M., Khan Y.H., Hafeez S., Massoud E.E.S., Rahman M.H. (2022). Appraisal for the Potential of Viral and Nonviral Vectors in Gene Therapy: A Review. Genes.

[53] Ramamoorth M., Narvekar A. (2015). Non viral vectors in gene therapy- an overview. J. Clin. Diagn. Res..

[54] Ren S., Wang M., Wang. C., Wang Y., Sun C., Zeng Z., Cui H., Zhao X. (2021). Application of Non-Viral Vectors in Drug Delivery and Gene Therapy. Polymers.

[55] Kay M.A., Nakai H. (2003). Looking into the safety of AAV vectors. Nature.

[56] Lisowski L., Tay S.S., Alexander I.E. (2015). Adeno-associated virus serotypes for gene therapeutics. Curr. Opin. Pharmacol..

[57] Everson E.M., Trobridge G.D. (2016). Retroviral vector interactions with hematopoietic cells. Curr. Opin. Virol..

[58] Daya S., Berns K.I. (2008). Gene therapy using adeno-associated virus vectors. Clin. Microbiol. Rev..

[59] Shaw A., Cornetta K. (2014). Design and Potential of Non-Integrating Lentiviral Vectors. Biomedicines.

[60] Surace E.M., Auricchio A. (2008). Versatility of AAV vectors for retinal gene transfer. Vision. Res..

[61] Bulcha J.T., Wang Y., Ma H., Tai P.W.L. (2021). Viral vector platforms within the gene therapy landscape. Signal Transduct. Target. Ther..

[62] Sayedahmed E.E., Kumari R., Mittal S.K. (2019). Current Use of Adenovirus Vectors and Their Production Methods. Methods Mol. Biol..

[63] Scarsella L., Ehrke-Schulz E., Paulussen M., Thal S.C., Ehrhardt A., Aydin M. (2024). Advances of Recombinant Adenoviral Vectors in Preclinical and Clinical Applications. Viruses.

[64] Wang D., Tai P.W.L., Gao G. (2019). Adeno-associated virus vector as a platform for gene therapy delivery. Nat. Rev. Drug Discov..

[65] Wang J.H., Gessler D.J., Zhan W., Gallagher T.L., Gao G. (2024). Adeno-associated virus as a delivery vector for gene therapy of human diseases. Signal Transduct. Target. Ther..

[66] Arsenijevic Y., Berger A., Udry F., Kostic C. (2022). Lentiviral Vectors for Ocular Gene Therapy. Pharmaceutics.

[67] Berkowitz R., Ilves H., Lin W.Y., Eckert K., Coward A., Tamaki S., Veres G., Plavec I. (2001). Construction and molecular analysis of gene transfer systems derived from bovine immunodeficiency virus. J. Virol..

[68] Yáñez-Muñoz R.J., Balaggan K.S., MacNeil A., Howe S.J., Schmidt M., Smith A.J., Buch P., MacLaren R.E., Anderson P.N., Barker S.E. (2006). Effective gene therapy with nonintegrating lentiviral vectors. Nat. Med..

[69] Mitrophanous K., Yoon S., Rohll J., Patil D., Wilkes F., Kim V., Kingsman S., Kingsman A., Mazarakis N. (1999). Stable gene transfer to the nervous system using a non-primate lentiviral vector. Gene Ther..

[70] Ali R.R., Reichel M.B., De Alwis M., Kanuga N., Kinnon C., Levinsky R.J., Hunt D.M., Bhattacharya S.S., Thrasher A.J. (1998). Adeno-associated virus gene transfer to mouse retina. Hum. Gene Ther..

[71] Rolling F., Shen W.Y., Tabarias H., Constable I., Kanagasingam Y., Barry C.J., Rakoczy P.E. (1999). Evaluation of adeno-associated virus-mediated gene transfer into the rat retina by clinical fluorescence photography. Hum. Gene Ther..

[72] Dudus L., Anand V., Acland G.M., Chen S.J., Wilson J.M., Fisher K.J., Maguire A.M., Bennett J. (1999). Persistent transgene product in retina, optic nerve and brain after intraocular injection of rAAV. Vision. Res..

[73] Bennett J., Duan D., Engelhardt J.F., Maguire A.M. (1997). Real-time, noninvasive in vivo assessment of adeno-associated virus-mediated retinal transduction. Invest. Ophthalmol. Vis. Sci..

[74] Flannery J.G., Zolotukhin S., Vaquero M.I., LaVail M.M., Muzyczka N., Huswirth W.W. (1997). Efficient photoreceptor-targeted gene expression in vivo by recombinant adeno-associated virus. Proc. Natl. Acad. Sci. USA.

[75] Balaggan K.S., Ali R.R. (2012). Ocular gene delivery using lentiviral vectors. Gene Ther..

[76] Xu D., Khan M.A., Ho A.C. (2021). Creating an Ocular Biofactory: Surgical Approaches in Gene Therapy for Acquired Retinal Diseases. Asia Pac. J. Ophthalmol..

[77] Ding K., Shen J., Hafiz Z., Hackett S.F., Silva R.L.E., Khan M., Lorenc V.E., Chen D., Chadha R., Zhang M. (2019). AAV8-vectored suprachoroidal gene transfer produces widespread ocular transgene expression. J. Clin. Investig..

[78] Ross M., Ofri R. (2021). The future of retinal gene therapy: Evolving from subretinal to intravitreal vector delivery. Neural Regen. Res..

[79] Dalkara D., Byrne L.C., Klimczak R.R., Visel M., Yin L., Merigan W.H., Flannery J.G., Schaffer D.V. (2013). In vivo-directed evolution of a new adeno-associated virus for therapeutic outer retinal gene delivery from the vitreous. Sci. Transl. Med..

[80] Kansara V., Muya L., Wan C.R., Ciulla T.A. (2020). Suprachoroidal Delivery of Viral and Nonviral Gene Therapy for Retinal Diseases. J. Ocul. Pharmacol. Ther..

[81] Wykoff C.C., Avery R.L., Barakat M.R., Barakat M.R., Boyer D.S., Brown D.S., Brown D.M., Brucker A.J., Cunningham E. (2024). T Jr.; Heier, J.S.; et al. Suprachoroidal Space Injection Technique: Expert Panel Guidance. Retina.

[82] Chung S.H., Mollhoff I.N., Mishra A., Sin T.N., Ngo T., Ciulla T., Sieving P., Thomasy S.M., Yiu G. (2021). Host Immune Responses after Suprachoroidal Delivery of AAV8 in Nonhuman Primate Eyes. Hum. Gene Ther..

[83] Liu Y., Fortmann S.D., Shen J., Wielechowski E., Tretiakova A., Yoo S., Kozarsky K., Wang J., Wilson J.M., Campochiaro P.A. (2018). AAV8-antiVEGFfab Ocular Gene Transfer for Neovascular Age-Related Macular Degeneration. Mol. Ther..

[84] Marcus D. Suprachoroidal Delivery of Investigational ABBV-RGX-314 for Diabetic Retinopathy: The Phase II ALTITUDE Study Dose Levels 1 and 2: One Year Results. Proceedings of the Annual Meeting of the Macula Society.

[85] Calton M.A., Croze R.H., Burns C., Beliakoff G., Vazin T., Szymanski P., Schmitt C., Klein A., Leong M., Quezada M. (2024). Design and Characterization of a Novel Intravitreal Dual-Transgene Genetic Medicine for Neovascular Retinopathies. Invest. Ophthalmol. Vis. Sci..

[86] Sheth V. SPECTRA: DME Part 1 32 Week Interim Data. https://ir.4dmoleculartherapeutics.com/static-files/15cda318-ccbf-4b58-81bc-2ecdb4d4907d.

[87] Gelfman C.M., Grishanin R., Bender K.O., Nguyen A., Greengard J., Sharma P., Nieves J., Kiss S., Gasmi M. (2021). Comprehensive Preclinical Assessment of ADVM-022, an Intravitreal Anti-VEGF Gene Therapy for the Treatment of Neovascular AMD and Diabetic Macular Edema. J. Ocul. Pharmacol. Ther..

[88] Wykoff C.C. Intravitreal Gene Therapy for Diabetic Macular Edema with ADVM-022: First Time Data Presentation of Prospective, Randomized, Phase 2 INFINITY Trial. Proceedings of the Annual Meeting of the American Society of Retinal Specialists.

[89] Yi Y., Hahm S.H., Lee K.H. (2005). Retroviral gene therapy: Safety issues and possible solutions. Curr. Gene Ther..

[90] Raper S.E., Chirmule N., Lee F.S., Wivel N.A., Bagg A., Gao G.P., Wilson J.M., Batshaw M.L. (2003). Fatal systemic inflammatory response syndrome in a ornithine transcarbamylase deficient patient following adenoviral gene transfer. Mol. Genet. Metab..

[91] Wilson J.M. (2009). Lessons learned from the gene therapy trial for ornithine transcarbamylase deficiency. Mol. Genet. Metab..

[92] Marshall E. (1999). Gene therapy death prompts review of adenovirus vector. Science.

[93] Cavazzana-Calvo M., Hacein-Bey S., de Saint Basile G., Gross F., Yvon E., Nusbaum P., Selz F., Hue C., Certain S., Casanova J.L. (2000). Gene therapy of human severe combined immunodeficiency (SCID)-X1 disease. Science.

[94] Kaiser J. (2003). Gene therapy. Seeking the cause of induced leukemias in X-SCID trial. Science.

[95] Kohn D.B., Sadelain M., Glorioso J.C. (2003). Occurrence of leukaemia following gene therapy of X-linked SCID. Nat. Rev. Cancer.

[96] Purdy R., John M., Bray A., Clare A.J., Copland D.A., Chan Y.K., Henderson R.H., Nerinckx F., Leroy B.P., Yang P. (2025). Gene Therapy-Associated Uveitis (GTAU): Understanding and mitigating the adverse immune response in retinal gene therapy. Prog. Retin. Eye Res..

[97] Chan Y.K., Dick A.D., Hall S.M., Langmann T., Scribner C.L., Mansfield B.C., Ocular Gene Therapy Inflammation Working Group (2021). Inflammation in Viral Vector-Mediated Ocular Gene Therapy: A Review and Report From a Workshop Hosted by the Foundation Fighting Blindness, 9/2020. Transl. Vis. Sci. Technol..

[98] Peters S., Heiduschka P., Julien S., Ziemssen F., Fietz H., Bartz-Schmidt K.U., Schraermeyer U., Tübingen Bevacizumab Study Group (2007). Ultrastructural Findings in the Primate Eye After Intravitreal Injection of Bevacizumab. Am. J. Ophthalmol..

